# Supplementary material for: MoCA Domain-Specific Pattern of Cognitive Impairment in Stroke Patients Attending Intensive Inpatient Rehabilitation: A Prospective Study
Source: Behav Sci (Basel). 2024 Jan 9;14(1):42. doi: 10.3390/bs14010042 (PMC10813017; doi:10.3390/bs14010042)
Supplement: Supplementary file 1 [file behavsci-14-00042-s001.zip › behavsci-2720142-supplementary.pdf]

Supplementary materials

Table S1. Evaluation tools in the RIPS study.

Area of competence	*Evaluation tool*	*Acronym*
Clinical and nursing complexity	Cumulative Illness Rating Scale	CIRS
	Markers of complexity	/
	Functional Oral Intake Scale/water swallow test	FOIS
	Numeric Rain Scale/pain assessment in advanced dementia	NRS<br>PAINAD
	Hospital Anxiety Depression Scale	HADS
	Stroke Aphasia Depression Questionnaire	SADQ-10
Neurological profile	National Institutes of Health Stroke Scale	NIHSS
	Trial of Organization in Acute Stroke Treatment	TOAST
	Oxfordshire Community Stroke Project	OCSP
Functional evaluation	Trunk Control Test	TCT
	Fulg-Meyer Assessment scale	FMA
	Short Physical Performance Battery	SPPB
	Modified Barthel Index	MBI
	Modified Rankin scale	MRS
	Scale of disability in communication	SDC
	Functional Ambulation Categories	FAC
	Medical Research Council scale	MRC
	Activity of Daily Living disabilities: Frenchay Activity Index	FAI
	Activity of Daily Living disabilities: Functional Walking Categories	FWC
Neuropsychological evaluation	Montreal Cognitive Assessment	MoCA
	Oxford Cognitive Screening	OCS

Table S2. Comparisons among included and excluded samples.

Variables	*Included sample (N=67)*	*Excluded sample (N=167)*	*p-value*
Age (years)	76.0 [16.0]	82.0 [12.0]	**0.002**
Gender	Male: 35 (52.2%)<br>Female: 32 (47.8%)	Male: 81 (48.5%)<br>Female: 86 (51.5%)	0.665
Education (years)	8.00 [8.00]	8.00 [8.00]	0.211
Time from the event (days)	11.0 [9.00]	11.0 [9.0]	0.492
Type of stroke	Ischemic: 49 (73.1%)<br>Haemorrhagic: 18 (26.9%)	Ischemic: 136 (81.4%)<br>Haemorrhagic: 31 (18.6%)	0.213
Side of stroke	Right: 35 (52.2%)<br>Left: 24 (35.8%)<br>Bilateral: 6 (9.0%)	Right: 63 (40.6%)<br>Left: 82 (52.9%)<br>Bilateral: 10 (6.5%)	0.091
Area of the lesion	None: 4 (6.0%)<br>Supratentorial: 52 (77.6%)<br>Subtentorial: 8 (11.9%)<br>Both: 3 (4.5%)	None: 14 (8.4%)<br>Supratentorial: 132 (79.0%)<br>Subtentorial: 16 (9.6%)<br>Both: 5 (3.0%)	0.803
NIHSS score	5.00 [6.00]	8.00 [9.00]	**<0.001**
MBI score	37.0 [45.0]	19.0 [38.0]	**<0.001**
MoCA dichotomised	Normal: 34 (50.7%)<br>Altered: 33 (49.3%)	Normal: 19 (24.4%)<br>Altered: 59 (75.6%)	**0.003**
Length of stay (days)	32.0 [20.0]	30.5 [29.0]	0.358

Table S3. Description of medications and medical history at baseline.

Variables	*Included sample (N=67)*
Medications	
Antidepressive medications	Yes: 27 (40.3%)<br>No: 40 (59.7%)
Antiepileptic mediations	Yes: 10 (14.9%)<br>No: 57 (85.1%)
Anti-parkinsonian medications	Yes: 0 (0%)<br>No: 67 (100%)
Antidepressive and antiepileptic medications	Yes: 3 (4.5%)<br>No: 64 (65.5%)
Medical history (Cumulative Illness Rating Scale – CIRS)<br>***1: No problem***<br>***2: Minor current problem or significant history***<br>***3: Morbidity or moderate discomfort, requiring treatment***<br>***4: Severe problem, constant significant discomfort, chronic problem difficult to control***<br>***5: Extremely severe problem, requiring immediate treatment, organ failure or severe functional impairment***	

Cardiac	1: 37(55.2%)<br>2: 10 (14.9%)<br>3: 16 (23.9%)<br>4: 3 (4.5%)<br>5: 1 (1.5%)
Hypertension	1: 20 (29.9%)<br>2: 6 (9.0%)<br>3: 40 (59.7%)<br>4: 1 (1.5%)<br>5: 0 (0%)
Vascular	1: 42 (62.7%)<br>2: 5 (7.5%)<br>3: 17 (25.4%)<br>4: 2 (3.0%)<br>5: 1 (1.5%)
Respiratory	1: 57 (81.1%)<br>2: 7 (10.4%)<br>3: 2 (3.0%)<br>4: 1 (1.5%)<br>5: 0 (0%)
Oculistic	1: 57 (85.1%)<br>2: 7 (10.4%)<br>3: 2 (3.0%)<br>4: 1 (1.5%)<br>5: 0 (0%)
Gastroenteric inferior	1: 55 (82.1%)<br>2: 9 (13.4%)<br>3: 2 (3.0%)<br>4: 1 (1.5%)<br>5: 0 (0%)
Gastroenteric superior	1: 62 (92.5%)<br>2: 4 (6.0%)<br>3: 1 (1.5%)<br>4: 0 (0%)<br>5: 0 (0%)
Liver	1: 62 (92.5%)<br>2: 5 (7.5%)<br>3: 0 (0%)<br>4: 0 (0%)<br>5: 0 (0%)
Kidney	1: 61 (91.0%)<br>2: 6 (9.0%)<br>3: 0 (0%)<br>4: 0 (0%)<br>5: 0 (0%)
Genitourinary	1: 49 (73.1%)<br>2: 10 (14.9%)<br>3: 8 (11.9%)

	4: 0 (0%)<br>5: 0 (0%)
Muscles, bones, skin	1: 52 (77.6%)<br>2: 12 (17.9%)<br>3: 3 (4.5%)<br>4: 0 (0%)<br>5: 0 (0%)
Central and peripherical nervous system	1: 42 (62.7%)<br>2: 6 (9.0%)<br>3: 17 (25.4%)<br>4: 1 (1.5%)<br>5: 1 (1.5%)
Endocrine	1: 37 (55.2%)<br>2: 5 (6.0%)<br>3: 26 (38.8%)<br>4: 0 (0%)<br>5: 0 (0%)
Psychiatric	1: 54 (80.6%)<br>2: 4 (6.0%)<br>3: 9 (13.4%)<br>4: 0 (0%)<br>5: 0 (0%)
